# TRIM28 as a novel transcriptional elongation factor

**DOI:** 10.1186/s12867-015-0040-x

**Published:** 2015-08-21

**Authors:** Heeyoun Bunch, Stuart K Calderwood

**Affiliations:** Department of Radiation Oncology, Beth Israel Deaconess Medical Center, Harvard Medical School, Center for Life Sciences, 3 Blackfan circle, Boston, MA 02115 USA

**Keywords:** Tripartate 28, Regulation, Transcription, Elongation, Phosophorylation

## Abstract

TRIM28 is a multidomain protein with versatile functions in transcription and DNA repair. Recently it was shown that this factor plays unanticipated roles in transcriptional elongation. TRIM28 was shown to stabilize the pausing of RNA polymerase II (Pol II) close to the transcriptional start site in many unactivated genes, permitting Pol II accumulation and readying genes for induction. In addition, the factor was shown to respond rapidly to signals accompanying transcriptional activation permitting the productive elongation of RNA by previously paused Pol II. We discuss here critical regulatory mechanisms of TRIM28 in transcriptional control and DNA repair that may illuminate the novel roles of this factor in pausing and elongation of Pol II.

## Background

Transcription is one of the major cellular processes to access the genome and regulate gene expression. Finely controlled gene expression is crucial to determine cell identity and maintain normal cell growth and homeostasis. During transcription, the RNA polymerase II (Pol II) complex carries out the generation of messenger RNAs and the majority of non-coding genes in eukaryotic cells [1, 2], and depending on the functional status and position of Pol II, transcription has been studied in three stages: transcriptional initiation, elongation, and termination [3].

Transcriptional initiation is the initial checkpoint in gene expression, where Pol II and general transcription factors (GTFs) are recruited to the promoter region upon activation [4]. Then, once Pol II becomes triggered to escape from the promoter, it elongates a nascent RNA transcript before releasing the fully transcribed RNA strand and finally dissociating from the gene terminus [3]. However, recent genome-wide analyses have revealed an additional regulatory step situated between early and processive elongation. This new, prevailing mechanism of regulation in metazoans, especially for developmental and stimulus-inducible genes, is called Pol II promoter-proximal pausing, in which Pol II is already engaged with TSS between +20 and +100 before transcriptional activation [5–8]. This TSS-bound, paused yet active Pol II has the capability to resume transcription upon receipt of activating signals. Approximately 30% of all protein coding genes displays Pol II paused in the promoter-proximal region [8]. Thus Pol II pausing has been recognized as another major checkpoint along with transcriptional initiation for gene activation [8, 9]. As a newly emerging, regulatory mechanism for transcription, Pol II promoter proximal pausing is undergoing rigorous investigation. A number of protein factors have been identified as regulators of Pol II pausing. NELF (negative elongation factor), DSIF (DRB sensitivity inducing factor), and POLR2M (DNA directed RNA polymerase II subunit) induce and stabilize Pol II pausing while positive transcription elongation factor (P-TEFb), MYC, ELL, TFIIS, CDK8-Mediator, and TFIIF facilitate Pol II pause release and entering into processive elongation [5, 6, 10–15]. In addition, we have recently discovered a novel role for the factor TRIM28 in the control of pausing of Pol II in mammalian genes genome-wide, a mechanism that is the subject of discussion here [16].

## TRIM28, a multi-domain protein

TRIM28 (TRIpartate motif-containing protein 28), also known as KAP1 (KRAB-associated protein 1) and transcription intermediary factor 1β (TIF1β), was first discovered as a polypeptide interacting with zinc finger family members of the Kruppel transcription factor family (KRAB) [17–21]. (For a comprehensive review on TRIM28, readers are directed to Iyengar et al. [22]). TRIM28 was initially shown to function alongside KRAB factors in gene repression [22]. This factor has subsequently been shown to be a highly versatile multidomain protein that is found associated with many genes throughout the genome.

**Figure 1:**
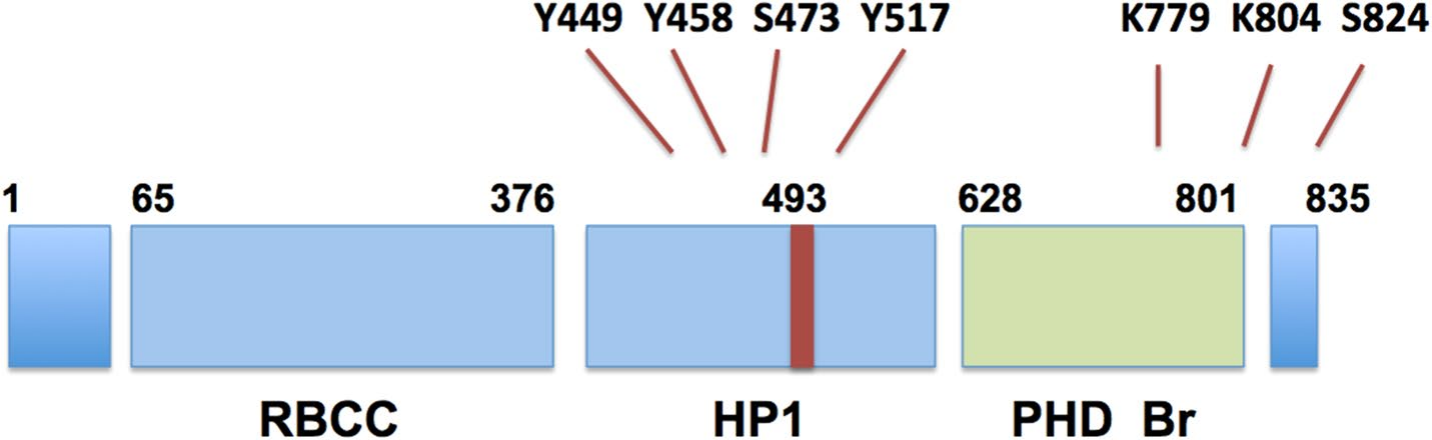
Major functional domains and key sites for PTM in TRIM28. The factor contains multiple functional domains. TRIM28 binds to KRAB transcription factors through the RBCC domain in the N-terminal portion and to heterochomatin 1 (HP1) proteins through a centrally located binding motif. Gene repression is mediated through C-terminal domains including adjacent PHD and BR domains. Key posttranslational modifications (PTM) include tyrosine and serine phosphorylation sites clustered around the HP1 binding motif and sumoylation sites in the BR domain. A critically important regulatory phosphorylation site is serine 824 located in the extreme C-terminal region.

**Fig. 2:**
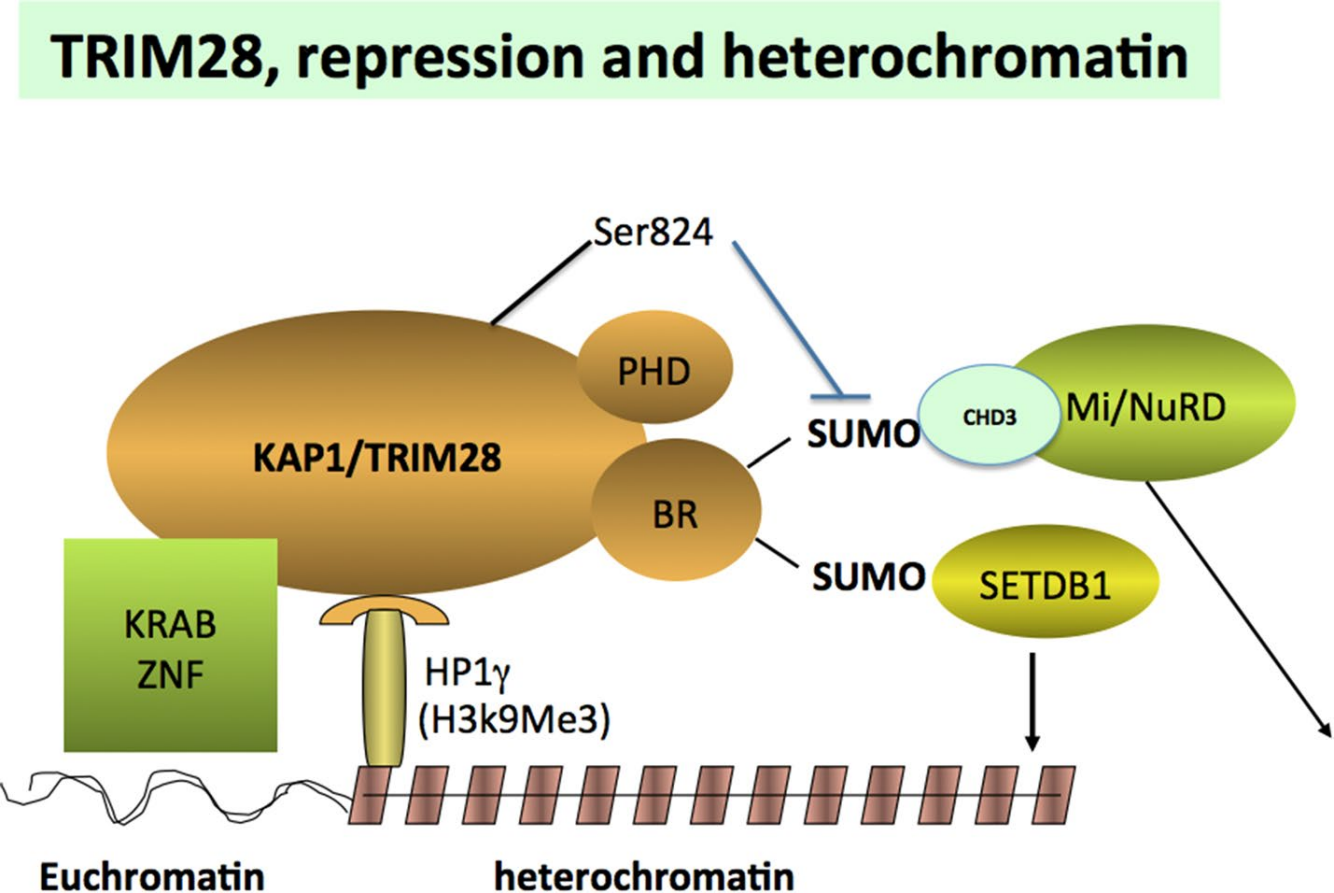
Representation of mechanisms of association of TRIM28 with chromatin and influences in transcriptional function. TRIM28 is depicted as associating with KRAB factors through the RBCC domain and with HP1 factors associated with H1K9Me3 though the HP1 binding motif. The sumoylated BR domain is represented as interacting with both CHH3/Mi2/NuRD complexes and with SETDB1 and mediating trans-repression.

TRIM28 was shown to contain an N terminal RBCC domain that is comprised of a RING (really interesting new gene) finger, two B-box zinc fingers and a coiled coil domain through which it interacts with KRAB proteins and is recruited to DNA [17, 23–25] (Figure 1). The RING motif is highly represented among mammalian proteins and exhibits ubiquitin E3 ligase activity [18, 26]. Adjacent to the RBCC domain is the short TIF1 signature domain that is essential for gene repression [23]. At the C-terminus are two adjacent domains with key roles in *trans*-repression of target genes. These are the PHD (plant homeo domain) and the C-terminal BR (BRomo domain). The BR domain of TRIM28 is atypical in that it does not bind to acetyl lysine residues [27]. The PHD domain possesses E3 ligase activity and can lead to multiple modifications on the BR domain by SUMO addition [28–30]. Sumoylation then “arms” the BR domain for interaction with mediators of repression, permitting it to associate with the SUMO interaction (SIM) domains in Mi2/NuRD complexes (with repressive HDAC activity) and with SETDB1 a histone methyltransferase that leads to trimethylation of histone H3 on lysine 9 (HeK9) on chromatin [31] (Figures 1, 2). H3K9Me3 is a classic mark of silent heterochromatin. TRIM28 is closely associated with regions rich in H3K9 in the genome [22]. However, TRIM28 has not been reported to bind directly to DNA. This factor has however been shown to be tethered to chromatin by association with KRAB factors through the RBCC domain [32]. In addition, TRIM28 contains a central binding site for HP1 (heterochromatin protein 1) and the factor is found associated with HP1 and H3K9 in areas of heterochromatin (Figures 1, 2) [33].

More recently, another level of regulation for TRIM28 involving phosphorylation has been discovered during investigation of the role of TRIM28 in DNA repair (see below). TRIM28 was shown to be recruited to the region of DNA double strand breaks in association with HP1 and to be rapidly phosphorylated close to the C terminus (on S824) by the DNA damage response kinase ATM (Figure 1) [34–36]. Phosphorylation on S824 led to loss of the SUMO residues within the BR domain, dissociation from NuRD and SETDB1 repressor complexes and accompanying relaxation of heterochromatin permitting DNA repair [35]. There thus appeared to be cycle of SUMO- and phospho-S824 modifications that governed cycles of contrasting TRIM28 activity [35]. A further wrinkle to this regulatory pathway was provided by findings that members of the Src family of non-receptor tyrosine kinases could suppress ATM-mediated TRIM28 modification and, during DNA repair, signal the termination of DNA damage mediated checkpoint signaling [37].

TRIM28 phosphorylation by such kinases, particularly Src itself, at multiple tyrosine residues (Y-449/Y-458/Y-517), located close to the HP1 binding-motif inhibited association of the factor with HP1 and reversed gene silencing mediated through binding to HP1 (Figure 1) [38]. Another phosphorylatable residue adjacent to the HP1 box, S473 was also associated with inhibition of TRIM28-HP1 binding and a decline in the intensity of the DNA damage response (Figure 1) [39]. Phosphorylation on this residue by DDR kinase Chk2 downstream of ATM led to both loss of repressor function in TRIM28, but also permitted binding to the factor E2F1 [40]. Thus negative charge introduced close to the HP1 box in TRIM28 appeared inhibitory to HP1 mediated-events, indicating another level of regulation by phosphorylation.

## Key role for TRIM28 in DNA repair

Understanding of the role of TRIM28 in pausing may be informed by current knowledge of its functions in DNA repair. White et al. and others have shown an important role for TRIM28 in DNA repair mechanisms [34, 35, 41]. These responses to DNA damage involved a role for the DNA damage response (DDR) kinase ATM in phosphorylating TRIM28 on S824 and presumably loss of the key Sumo residues from the BR domain [41]. Active, sumoylated TRIM28 was shown to bind rapidly to damaged chromatin in association with HP1, followed by phosphorylation on S824 and reversal of the silencing effects of the factor. In addition, resolution of the DSB response appeared to involve the phosphorylation of TRIM28 in residues adjacent to the HP1 box by Src family kinases as discussed earlier [35, 37]. Thus phosphorylation may counteract the repressive influence of TRIM28 by both reversing sumoylation of the BR domain and reducing association with HP1. Phosphatases also played a role in this response and PP1β was shown to interact with the coiled coil domain of TRIM28 followed by dephosphorylation of S824 and promoting DDR signaling [35]. Likewise PP4 could lead to dephosphorylation of S824 and was shown to play a key role in non-homologous end joining repair [42–44]. The role of TRIM28 in DDR signaling was recently attributed to activation of the histone acetylase Tip60 [45]. A complex containing TRIM28, HP1 and the histone methyltransferase suv39.h1 was shown to become associated with chromatin after DNA damage and led to cycles of histone H3K9 methylation and further binding of the TRIM28, HP1 and suv39.h1 to the H3K9Me regions [45]. This reaction was shown to create areas of acetylated H3K9 that could activate Tip60 and this led to acetylation and activation of ATM and modification of histone H4 by acetylation. This was shown to be a self-limiting interaction and TRIM28 phosphorylation by the activated ATM on S824 attenuated the response [45]. Overall the exact role of the rapid changes in histone H3K9 methylation in the response is not clear but these events did appear to play key roles in DDR signaling as well as in chromatin remodeling interactions that might be key to the access of repair proteins to areas of DNA damage [46–48].

## TRIM28 and transcriptional elongation

TRIM28 has been shown to be a powerful gene repressor when overexpressed in cells [31, 49]. This factor bound tightly to the 3′ region of members of the ZNF family in association with SETDB1 and chromatin areas rich in H3K9Me3, implying the establishment of a repressive transcriptional environment [32, 50]. In another study, TRIM28 was shown to bind TSSs of over 3,000 genes in mouse embryonic stem cells [51]. However, a clear role for TRIM28 in the transcriptional regulation of these genes was not established [32, 50]. In addition, TRIM28 was also shown to bind to the promoter regions of a number of genes, interestingly, independently of the RBCC domain. Iyengar et al. [32] showed that such recruitment involved protein–protein interactions in a central (380–618) region of TRIM28 independent of the HP1 box. The implications of such interactions seemed however unclear.

Recently, in an unbiased screen for proteins that bound at the pausing site to regulate Pol II pausing on the human *HSP70* (*HSPA1B*) gene, TRIM28 was identified [16]. TRIM28 was found associated with the non-template DNA of *HSPA1B* close to the transcriptional start site (TSS) at around +70. Using an in vitro transcription assay, it was then shown that TRIM28 could stabilize pausing of Pol II on *HSPA1B* and that depletion of the factor from HeLa nuclear extracts used in the assay led to increased transcriptional elongation [16]. It could thus be predicted that reduction in TRIM28 levels would lead to increases in basal level of productive elongation and gene expression of *HSPA1B.* Indeed, knockdown of TRIM28 led to increases in *HSPA1B* RNA and protein levels in vivo as well as levels of other proteins regulated by Pol II pausing such as *NFB* and *ERK1* [16]. ChIP-seq studies of Pol II occupancy in murine ES cells with or without knockdown of TRIM28 reinforced the function of TRIM28 in regulating Pol II pausing. Pausing indices were analyzed as the ratio of promoter proximal Pol II (−250 to +250 from TSS) to elongating Pol II defined here as gene body Pol II (+500 to +2,500 or the gene end). TRIM28 knockdown modulated pausing index in a large number of genes many that had been shown previously to be regulated by Pol II promoter-proximal pausing. These included the *HSPA1B*, *ERK1*, *JUN* and *EGR1* genes [16]. These data therefore indicated a commanding role for TRIM28 in regulating Pol II pausing and pause release.

**Figure 3:**
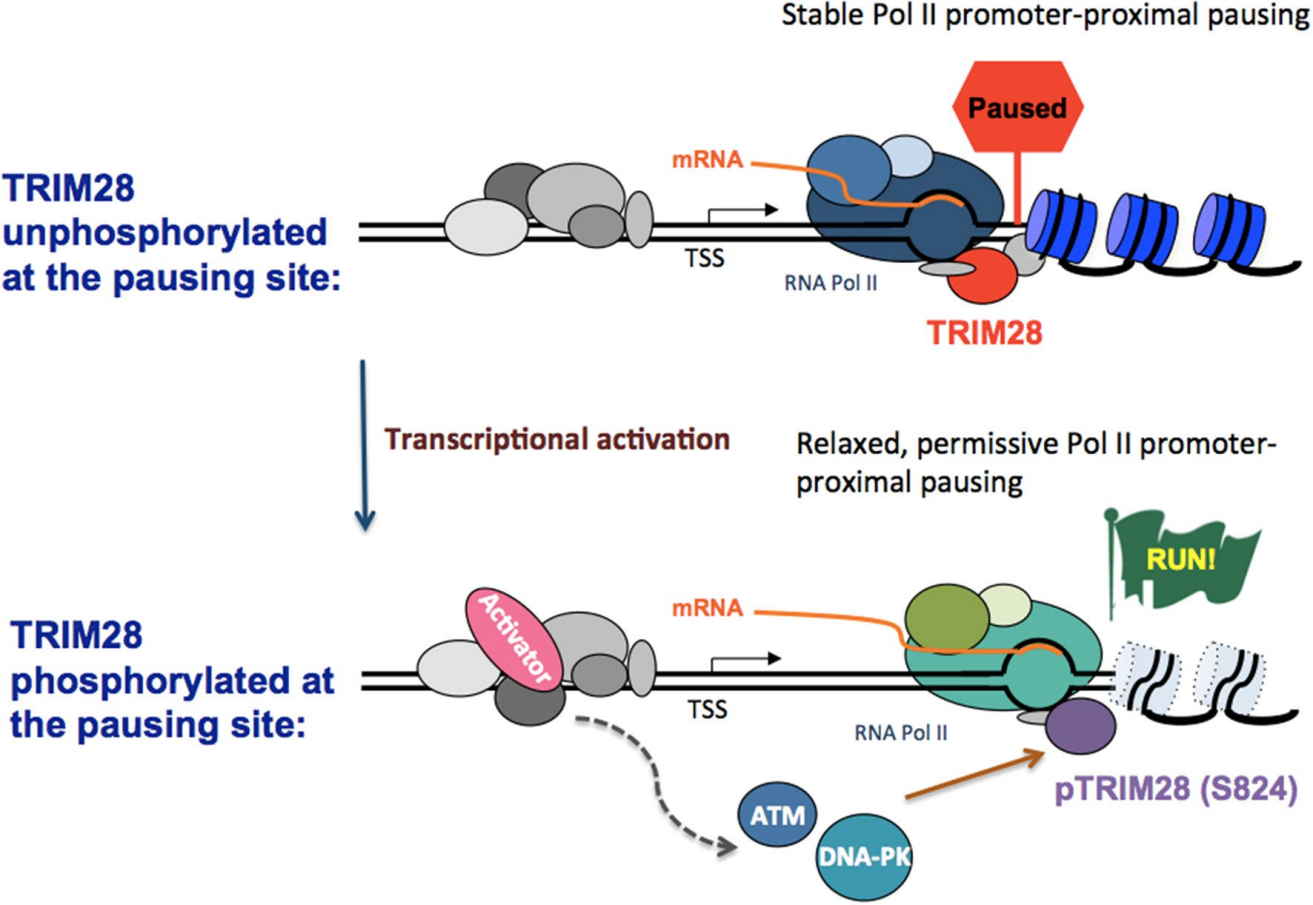
A model of TRIM28-mediated transcriptional regulation in Pol II promoter-proximal pausing and pause release. A transcriptional repressor TRIM28 is bound adjacent to the Pol II pausing site to stabilize the paused complex of Pol II. In a model paused gene, HSPA1B, heat-shock instantly recruits a gene-specific transcriptional activator, HSF1 to activate transcription. Upon transcriptional activation, TRIM28 is rapidly phosphorylated at S824. This phosphorylation is dependent on ATM and DNA-PK, critical DNA damage and repair kinases. This phosphorylation signaling appears important for Pol II pause release as blocking the function of these kinases interferes with Pol II progression into the 3′ terminus of HSPA1B.

The next question was: how could TRIM28-mediated Pol II pausing be overturned in vivo after transcriptional activation? One possibility considered was that TRIM28 could dissociate from the promoter proximal site after transcriptional activation. However, ChIP-qPCR experiments carried out on *HSPA1B* during heat shock showed little evidence of TRIM28 release [16]. Taking a lesson from p21 transcription regulated by TRIM28 phosphorylation [35], it was found that the factor became rapidly phosphorylated on S824, within seconds of heat shock, a time when *trans*-activator HSF1 was shown to bind to *HSP* genes [16, 34, 41]. Next, kinases potentially involved in S824 phosphorylation were examined. DNA-dependent protein kinase (DNA-PK) kinase was investigated since TRIM28 had been shown to interact with the DNA-PK catalytic subunit and its regulatory subunit Ku70 in immunoprecipitation experiments followed by mass spectrometry analysis [16]. ATM was studied due to its known involvement in TRIM28 S824 phosphorylation after DNA damage and for its overlapping functions and substrates with DNA-PK. It was found that inhibition of DNA-PK as well as ATM kinase activity inhibited the phosphorylation of TRIM28 on S824 [16]. Significantly, inhibiting these kinases dramatically reduced Pol II occupancy in the gene terminus when transcription was activated in *HSPA1B*, suggesting an important role of this phosphorylation signaling on transcriptional elongation. The significance of TRIM28 phosphorylation in Pol II pause release was confirmed in in vitro transcription experiments showing that phosphomimetic mutation of S824 by aspartate substitution abolished the ability of TRIM28 to mediate Pol II pausing on *HSPA1B.* These findings established a role for TRIM28 in Pol II pausing regulation and a mechanism for pausing release involving DNA damage-triggered kinases DNA-PK and ATM. In this model, unphosphorylated TRIM28 stabilizes Pol II pausing at the pausing site. Upon transcriptional activation, ATM and DNA-PK become activated to phosphorylate TRIM28 at S824, potentially leading to more favorable nucleosome architecture for Pol II processive elongation (Figure 3).

## General discussion

These studies open up a wide range of issues for discussion and further experimentation. For instance, the finding that S824 phosphorylation reverses the influence of TRIM28 on pausing might suggest that Pol II pausing requires the active poly-sumoylated form of the BR domain. This finding would also implicate potential roles for SETDB1 and the Mi2/NuRD complex in pausing. Indeed, one hypothesis for the establishment of stable transcriptional pausing is that this mechanism might be influenced by the positioning of the first nucleosome in the gene body [8, 9]. One might thus suggest a role for H3k9Me3 modification of such structures and association of TRIM28 with such structures through its HP1 binding domain in maintenance of Pol II pausing.

Another question is—how is this novel mechanism involving TRIM28 to be dovetailed with established pathways for regulating Pol II pausing? The principle mechanism for mediation of pausing involves the factors NELF and DSIF that mediate arrest of Pol II until transcriptional stimulus. Transcriptional activation involves recruitment of the kinase complex P-TEFb to the activated gene, phosphorylation of NELF and serine 2 on the C-terminal repeat sequence of Pol II (Pol II phospho-S2) by P-TEFb, disengagement of NELF, and subsequent elongation [9]. ChIP Seq studies indicated a significant increase in the levels of Pol II phospho-S2 in the gene bodies of a large number of genes when TRIM28 was knocked down [16]. It remains to be determined whether these findings indicate a primary role for TRIM28 in influencing Pol II phosphorylation at the S2 position or whether Pol II modification occurs indirectly as elongation is unleashed following *trans*-activation by inducing factors.

Many unresolved questions await further experimentation regarding TRIM28-mediated Pol II pausing regulation. For instance, a mechanism for the activation of the PIKK kinases ATM and DNA-PK prior to phosphorylation of TRIM28 at S824 needs to be established. How such principle signaling molecules in the DDR response could become activated in productive elongation is not clear. Previous studies of *trans*-activation in androgen receptor and estrogen receptor-regulated genes have shown association of target genes with the catalytic subunit of DNA-PK, DNA-PK associated proteins Ku70 and Ku80, ATM, topoisomerase II and DNA repair intermediate poly (ADP-ribose polymerase (PARP1), an association leading to transcription through a mechanism that may involve generation of DNA double strand breaks in the activated gene. [52–54]. In addition, DNA-PK was shown, in previous studies to associate directly with HSF1, the transcriptional regulator of *HSP* genes [55, 56]. Previous studies by the Lis lab showed that elongation was associated with processive movement of PARP1 into the gene body of *HSP70* in heat shocked cells and subsequent processive modification of histones by ADP ribosylation in *Drosophila*. This effect was triggered by HSF induced recruitment of Tip60 and histone acetylation on histone H2A, an effect required for activation of PARP1 residues pre-existing at the 5′ of the unactivated gene and triggering *HSP70* transcription [57]. These findings are reminiscent of the DNA repair studies mentioned above where exposure to DNA double strand breaks led to association of TRIM28 and HP1 with areas of H3K9Me3 on damaged chromatin that could activate Tip60 and lead to acetylation and activation of ATM [45].

Another question is—by what mechanism does TRIM28 interact with the TSS of the gene bodies of paused genes? TRIM28 may operate in pause regulation in a “hit and run” manner or might associate stably with chromatin. As mentioned earlier, it was shown that TRIM28 could associate with target areas of chromatin through: (1) binding to KRAB transcription factors through its RBCC domain [22], (2) association with methylated histones through HP1 binding to the HP1 binding motif [22], and (3) though a central domain remote from the HP1 box shown to bind unknown factors in gene promoters [32]. It is notable that HP1 has been reported to function in transcriptional elongation and to interact with the factor facilitates chromatin transcription (FACT) [58, 59]. Another possibility could be binding of TRIM28 to other transcription factors through the coiled-coil domain [22]. Mechanisms involving phosphorylation of the central region by nuclear tyrosine kinases discussed above, as observed in DNA repair studies, could be involved in regulating TRIM28 association with transcriptionally paused genes (Figure 1). In addition, it will be important to understand how nucleosome structures might be modified or changed by TRIM28 phosphorylation during Pol II pause release.

In conclusion therefore, TRIM28 appears to play a unique and essential role in transcriptional elongation. We anticipate future investigation of upstream and downstream signaling and the regulatory mechanisms that underlie the role of TRIM28 in transcriptional pausing and elongation.

## Abbreviations

TRIM28: TRIpartate motif-containing protein 28
KAP1: KRAB-associated protein 1
P-TEFb: positive transcription elongation factor
SETDB1: SET domain, bifurcated 1
NuRD: nucleosome remodeling deacetylase
DNA-PK: DNA dependent protein kinase
ATM: ataxia-telangiectasia mutated
